# Variability of serum oxidative stress biomarkers relative to biochemical data and clinical parameters of glaucoma patients

**Published:** 2010-07-09

**Authors:** Kaya N. Engin, Bülent Yemişci, Ulviye Yiğit, Ahmet Ağaçhan, Cihan Coşkun

**Affiliations:** 1 Clinics of Eye Diseases, Bağcılar Training and Research Hospital, Istanbul, Turkey; 2 Clinics of Eye Diseases, Bakırköy Şadi Konuk Training and Research Hospital, Istanbul, Turkey; 3 Clinics of Biochemistry, Bağcılar Training and Research Hospital, Istanbul, Turkey

## Abstract

**Purpose:**

The importance of oxidative stress in both the formation and the course of glaucoma has been known. Among the antioxidants, vitamin E possesses the specific effects and regulatory mechanisms of a neurohormone. The serum oxidant/antioxidant profile is reportedly altered in ocular pathologies. In this study, we analyzed the effect of the clinical parameters of glaucoma and biochemical data on antioxidants and serum oxidative stress markers as oxidation degradation products.

**Methods:**

In this multicenter case control study, control and patient groups consisted of 31 healthy individuals and 160 glaucoma patients with no known additional abnormalities, respectively. We analyzed the oxidation degradation products malonyl dialdehyde (MDA), advanced oxidation protein products (AOPP), antioxidants, vitamins E and A, Serine (Ser), superoxide dismutase (SOD), glutathione peroxidase (Gpx), transferrine (TF), and total antioxidant capacity (TADA). All of these parameters and their relationships with serum cholesterol, glucose, protein, albumin, triglyceride levels, age, gender, visual acuities, intraocular pressure (IOP), c/d ratio, gonioscopic findings, medications, presence of pseudoexfoliation (px), central visual field and Optical Coherence Tomography (OCT) data, pachymetry, and Laplace values, were evaluated individually. Statistical comparisons were performed among them, and with the control group as well.

**Results:**

TADA, AOPP, SOD, and Gpx were found to be decreased, and MDA, Ser, TF, vitamins A and E increased in the patient group. All data, excluding AOPP, varied significantly. Vitamin E was the most consistent parameter.

**Conclusions:**

In this study, the association between glaucoma and lipid oxidation was shown on a systematic basis, and the significance of vitamin E as a neuroprotective agent has been revealed once more.

## Introduction

Although glaucoma (recognized as the most frequent cause of irreversible blindness), is characterized by progressive retinal ganglion cell loss, it was regarded for years as “a disease associated with an increase in the intraocular pressure (IOP)” [1]. Even if increased IOP has been excluded from the definition of glaucoma, considered a major risk factor, and glaucoma has been defined as an optic neuropathy, current clinical applications strongly aim to decrease IOP. Though we have effective medical and surgical therapies at hand, progressive visual loss is still a prevalent symptom in glaucoma cases [2]. In light of current knowledge, a valid hypothesis is that ganglion cell death (apoptosis) observed in glaucoma is caused by a special type ischemia. Indeed, the clinical manifestations of glaucoma are different from other ischemic pathologies. Beyond animal experiments, ischemia and glaucoma can be induced by an increase in IOP, and quite different abnormalities have been observed using various methods, such as carotid occlusion [3]. In another study, the role of oxidative damage in the etiopathogenesis of glaucoma was explained by the production of reactive oxygen species secondary to a complex trabecular mitochondrial defect [4].

Tissue damage due to oxidation is a chain reaction, which is mainly initiated by the production of free oxygen radicals. Though these molecules interact with intracellular signal conduction and regulation mechanisms, they demonstrate their main effects as destructive changes induced by a series of DNA reactions and macromolecules, such as proteins and lipids [5]. Oxidation degradation products, which are tissue and serum markers of this destructive process, consist of malonyl dialdehyde (MDA), advanced lipid oxidation end-products (ALEs) for lipids, and advanced oxidation protein products (AOPPs) for proteins [6]. Vitamins E, C, and A, molecules like homocysteine, and transferrine (TF) bind oxygen ions and transform into steady-state compounds with their resultant antioxidant effects. Serine (Ser) is an amino acid used in the effect pathway of vitamin E. These buffer compounds that are formed offer their ions to the downregulating (velocity-limiting) systems, which consist of superoxide dismutase (SOD) and glutathione peroxidase (Gpx), to curtail chain reactions [7]. The nervous system, which also includes retina ganglion cells, is rich in lipids. Further, metabolic rate, oxygen degradation, and synthesis of ATP are increased in these cells, while the cellular regeneration rate is restricted. Dopamine oxidation and chemical factors (e.g., glutamate) are also important. Secondary to all of these factors, nerve cells are quite sensitive to oxidative damage [5].

The use of antioxidants for the prevention of glaucomatous decay is also addressed. Higher lipid contents of nerve cells has enhanced the importance of lipid-soluble vitamin E, especially α-tocopherol, which has hormone-like regulatory mechanisms with its unique transporter proteins and receptors, exerting neuromodulatory effects on the eye and other tissues. Neuroprotective effects of vitamin E compounds in retinal diseases and glaucoma have been clincally demonstrated [8]. Ginkgo biloba extracts are also neuroprotective antioxidants [9]. Both vitamin E compounds [10], and ginkgo biloba extracts [11] also manifest vasoregulatory activities in the retina, which are significant for the prevention of ischemia.

As a form of optic neuropathy, glaucoma has also demonstrated central nervous system pathologies in experimental [12], and clinical [13,14] studies. In addition to the pathogenesis of all types of glaucoma, oxidative damage plays a key role [5,15]. In our study, we investigated the effects of the clinical parameters of glaucoma, and relevant biochemical parameters on various oxidative stress indicators, such as increments in various antioxidants and oxidative degradation products in serum samples.

## Methods

Control and patient groups consisted of 31 and 160 individuals, respectively (Table 1). With routine examination, there were no findings implying ophthalmic pathologies, including glaucoma or ocular hypertension, in the control group. Patients with no known ocular or systemic concomitant disorders, previous glaucoma surgeries, and antioxidant usage, who received follow-up in our glaucoma polyclinics, were selected for the patient group. For both groups, oxidation degradation products (MDA and AOPP), antioxidants (e.g., vitamins E and A), Gpx, and total antioxidant capacity (TADA) were studied, in addition to routine blood biochemical tests for cholesterol, glucose, protein, albumin, and triglyceride. All of these parameteres with their relationships to blood cholesterol, glucose, protein, albumin and triglyceride levels, age, gender, visual acuity, intraocular pressure, c/d ratio, gonioscopic findings, drugs used, the presence of pseudoexfoliation (px), central field of vision, Mean deviation (MD)- Pattern Standart Deviation (PSD), pachymetry, and eye wall stress (Laplace’s value), were evaluated individually. The patients were examined on the day of blood sample collection. The patients with visual acuities less than 5/10 in one eye were considered to have lower visual acuity. IOPs and c/d ratios were taken with the Pascal Dynamic Contour tonometry (Nidek Inc., Fremont, CA) and RTVue-100 fourier domain Optical Coherens Tomography (OCT) (Nidek Inc.), respectively. Patients with IOPs higher than 21 mmHg in one or both eyes were evaluated as mono-or bilateral higher IOP groups, respectively, while those with c/d ratios more than 0.5 in one or both eyes were assessed as mono-or bilaterally increased IOP groups, respectively. Visual fields were taken with the Humphrey Field Analyzer Model 740i (Carl Zeiss Inc. Dublin, CA). Patients with glaucomateus visual field defects in one or both eyes were evaluated as mono-or bilateral visual field defect groups, respectively.

**Table 1:** Comparison of control and patient groups.

**Parameters**	**Control group**	**Patient group**
Mean age	44.87±10.78	50.96±14.19
Gender (F/M)	15/16	106/54
Cholesterol	173±46.67	201.23±43.25
Glucose	86±5.66	119.76±58.54
Triglyceride	118±38.18	170.46±86.84
Protein	0.78±7.95	8.05±0.6
Albumin	4.67±0.21	4.71±0.25

Corneal thickness <555 or >558 constituted groups with thinner or thicker corneas, respectively [16]. In gonioscopic examination, patients with grade ≤2 consisted of narrow-angle glaucoma patients, and the patients were divided into those using single (prostaglandin analogs, beta blockers), 2 and 3 drops, or patients without medications. Prostaglandin analoges and beta blockers were included in all of the combinations, and fixed combinations were considered as single drops. Corneal thickness of the patients were measured with the Pocket II pachymeter device (Quantel medical inc. Bozeman, MO). Axial diameters of corneas were measured with the AB5500+ (Sonomed Inc., L Success, NY) A scan mode, and together with the results of the pachymetre, the Laplace formula was applied [17]. Patients with IOPs higher or lower than normal eye wall stress values constituted higher and lower Laplace groups.

These results were compared statistically among one another, as well as with those of the control group. When sample sizes in all groups were appropriate for parametric tests, the Student *t*-test and Mann–Whitney U tests were used. Test results were evaluated as moderately (p<0.01), highly (p<0.005), or extremely (p<0.001) significant.

### Biochemical analyses

Routine biochemical analyses for serum glucose, cholesterol, triglyceride, total protein, and albumin were performed using the Roche autoanalyser DP (Roche Diagnostics Ltd. W Sussex, UK) modular calorimetric analytical method, while for other measurements were done manually.

#### Measurement of advanced oxidation protein products (AOPP)

AOPP levels were studied in the AU 2700 autoanalyser (Olympus Diagnostics Inc. Melville, NY). Blood samples were drawn from cubital veins in tubes with EDTA (EDTA), and centrifuged at 1660× g and 4 °C for 10 min. The blood samples were divided in aliquots and kept in Eppendorf tubes at −20 °C. All samples were analyzed within approximately 30 days. After preparation of chloramine-T stock solution (10 mmol/l), they were diluted 100 times with PBS (20 mmol/l, pH:7.4) to obtain a main standard solution at 100 µmol/l concentration (standard 1). Chloramine-T main standard solution (standard 1:100 µmol/l) was diluted with PBS (20 mmol/l, pH 7.4) to get a 5-point calibration curve, and lso to prepare Chloramine-T standards at 75, 50, 25, and 12.5 µmol/l concentrations. PBS (160 µl) was added to 40 µl standard or plasma, mixed, and incubated for 25 s. The absorbance of the mixture was read at 340 nm, then 20 µl of acetic acid was added and incubated for 25 s. Finally, 10 µl KI solution was added and reincubated for 25 s, and its absorbance was read again. All steps were completed at 37 °C in a single cuvette. Time intervals were arranged at every step as 25 s or longer based on the program characteristics of the analyzer. A calibration curve was formulated using absorbance (A) values corresponding to the concentrations of 5 standard solutions. AOPP concentration was reported as µmol Chloramine-T/l, corresponding to the absorbance measured.

#### Measurement of malonyl dialdehyde (MDA)

Blood samples drawn from the cubital vein were placed in blood tubes containing EDTA as an anticoagulant. Plasma samples were separated rapidly, and cryopreserved at −70 °C Samples were not thawed and refrozen, and they were also not exposed to light to avoid photooxidation. A 140 µl standard, sample, and reagent blank, were placed individually into microcentrifuge tubes. “Reagent“ (455 µl) was then added into each tube and vortexed. HCl (105 µl; 12 N [37%]) was added into each tube as well. The tubes were stirred throughly, tapped close, and incubated at 95 °C for 60 min in a milleu of acidity provided by HCl. Then, 1 molecule of MDA and 2 molecules of reagent (N-methyl-2-phenylenilindol) reacted with each other to yield a stable chromophor product (colored product), which might provide a maximal absorbance spectrum at a 586 nm wavelength. Centrifugation at 13,000× g for 15 min yielded a clear supernatant sample. This sample (150 µl) was placed in each well. Their fluorescent activities were measured on a microplate reader (Synergy^TM^ 2 Multi-Mode; BioTek Instruments, Inc., Vinooski, VT) at 500 nm (±30) excitation, and 586 nm (±30) emission. Using a y=ax+b formula derived from the absorbance-concentration correlation of standards used for the construction of the MDA standard curve, and absorbance data obtained, the analyzer automatically calculated MDA concentrations, and the results were expressed as “µmol/l.”

#### Measurement of TADA

Blood samples drawn from the cubital vein were taken into gelatinized tubes with no anticoagulant, centrifuged at 3,000× g, and 4 °C for 12 min, and their plasma components were separated into two aliquots. Dilution buffer, copper, and stop solutions were preserved at 2–8 °C, and working samples were kept as “aliquots” at −70 °C. Before measurements, dilution buffer, copper, and stop solutions were kept under room temperature for 30 min. To obtain a standard solution, 1.5 ml deionized water, and uric acid standard were added, and the prepared solution was vortexed until it dissolved thoroughly to obtain a “uric acid standard” with a final concentration of 2 nM.

From this stock standard solution, 5 working standards were obtained using serial dilutions. A dilution buffer was used in 1:4 dilutions of standard and sample solutions. Standard and sample solutions were diluted with the dilution buffer. Diluted standard, samples, reagent blank, and 200 µl buffer were pipetted into every well. The reagent blank contained buffer solution for dilution and the standard/sample solution. At 490 nm wavelength using a Roche COBAS MIRA Plus Chemistry Analyzer (Roche Diagnostics Ltd. W Sussex, UK), the absorbances of reagent blank, standard, and samples were read. Copper solution (50 µl) was added in each well, excluding wells containing reagent blank, incubated under room temperature for 3 min, and the reaction was finalized after adding 50 µl stop solution in each well. A second reading was done for absorbance at 490 nm wavelength. Under the combined effects of the sample of antioxidants in the standard solution, Cu^+^ (ferric form) in the copper solution is reduced into Cu^2+^ (ferro form), and a color reaction yielding maximal absorbance at 490 nm wavelength is formed. Using spectrophotometry, total antioxidant capacity (TAC) was automatically estimated based on the equation y=ax+b, derived from uric acid standard curve, and the results were recorded.

#### Determination of superoxide dismutase (SOD) activity

Blood samples were drawn from the cubital vein, placed into tubes containing EDTA as an anticoagulant, and centrifuged at 1,000× g and 4 °C for 10 min. Supernatant plasma was drawn using a pipette, and then discarded. The samples were mixed 4 times with ice water to disintegrate RBCs. A 250 µl sample and 400 µl of a ethanol/chloroform (62.5/37.5) solution were mixed to measure Cu-Zn SOD activity. Thus, inactivation of mitochondrial Mn-SOD and Fe-SOD by ethanol/chloroform mixture was achieved. Then, this mixture was centrifuged at 3,000× g and 4 °C for a minimum of 30 s. The samples were frozen at −70 °C, and analyzed within 1 month. The Roche COBAS MIRA Plus Chemistry Analyzer (Roche Diagnostics Ltd. W Sussex, UK) was used for manual measurement procedure. The results were evaluated in comparison with hemoglobin concentrations.

The determination of erythrocyte Gpx activity was achieved using a Cellular Glutathione Peroxidase kit (Cayman Chemical Ann Arbor, MI), modified for the Roche COBAS MIRA Plus analyzer (Roche Diagnostica). Gpx concentrations were calculated based on the Equation 1 mU/ml=1 nmol NADPH/ml/min=(A3, 40min)/0.00622. The results were evaluated in comparison with hemoglobin concentrations.

Transferrine (TF) analyses were performed using a Beckman kit.

Levels of vitamins E and A were measured using High Perfusion Lipid Chromatography (HPLC). The patient’s serum (200 µl) was placed into a tube and reacted with 200 µl (1 g/l) ethanol ascorbate to deproteinize. Asetonitrile (24 µl) was added to the mixture, and then vortexed for 1 min. Next, 500 µl of HPLC grade hexane was added to this solution, vortexed for 2 min, centrifuged at 770× g for 1 min,and a supernatant hexane phase was collected. This procedure was repeated 3 times. Vitamins A and E were derived from approximately 1,500 µl of hexane solution in these procedures. A completely clear 1,250 µl solution was drawn from the tube, removing a small amount of precipitated sediment that as present in the bottom of the tube. The hexane was completely evaporated under liquid nitrogen in a water bath at 36 °C. The tubes were closed watertight with paraffin, and prepared for the test. To perform the HPLC procedure, 10 µl (1 g/l) ethanol ascorbate was added to the sediment at the bottom of the tube, and then vortexed. Then, 150 µl of mobile phase (methanol:water; 95.5) was added, vortexed for 1 min, and degassed by sonication for 1 min. The mixture was sieved through a 45 nm filter. A 50 µl sample was removed from this filtered solution, injected into the HPLC Chrome-system column at a flow rate of 1.5 ml/min, and using the Knauer UV detector (Knauer Inc. Berlin, Germany), vitamins E and A were read at 295 nm and 320 nm, respectively. Peaks of vitamins A and E, which were derived individually from chromatograms obtained from the Spectra-Physics integrator (Triad Scientific Inc. Lakewood, NJ), were compared to calculate concentrations expressed as μg/ml.

## Results

**Figure 1:**
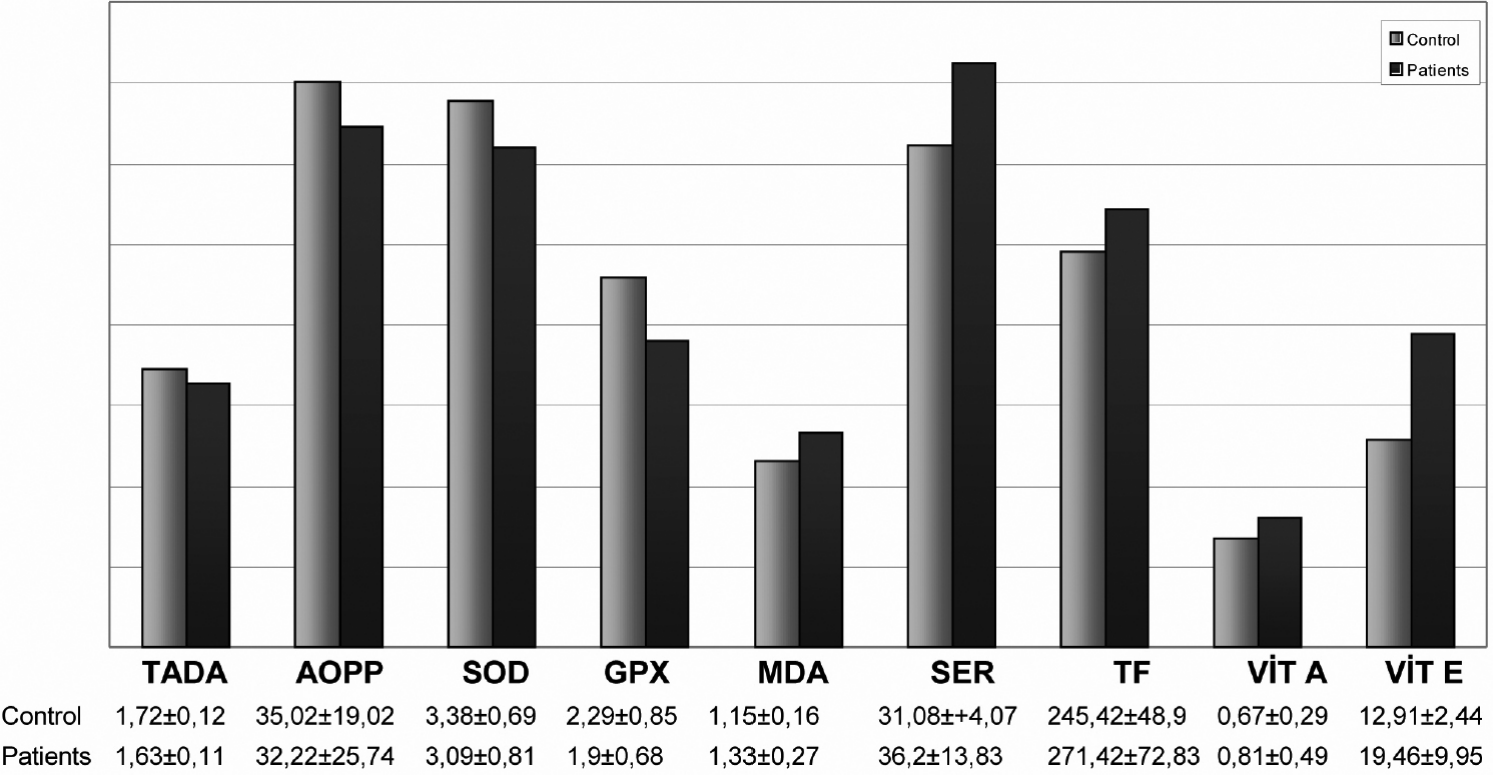
Comparison of the oxidative stress results between control and patient groups.

In comparison to the control group, TADA, AOPP, SOD, and Gpx were found to be decreased, while MDA, Ser, TF, and vitamins A and E increased in the patient group (Figure 1). Excluding AOPP, all data varied significantly.

For vitamin E and MDA, this increase was found to be extremely significant. In the hypertriglyceridemia group, MDA, vitamin E, TADA, Gpx, SOD, and TF levels varied significantly. In the hypo-and hyperproteinemia groups, MDA, and TADA levels varied significantly, and in the hypercholesterolemia group, all data (excluding AOPP) varied significantly. Vitamin E demonstrated extremely significant increases among all glycemia groups, in hypercholesterolemia, and hypertriglyceridemia, as well as in hyper-and normoproteinemia groups. In the hyperproteinemia group, an increase in Ser was found to be extremely significant. MDA increments were of utmost significance in the hypercholesterolemia group, while in the hypertriglyceridemia group, they were observed to be extremely significant (Table 2 and Table 3). The presence of px induces decreases in TADA and AOPP, and increases in MDA and vitamin E levels, while in the wide-angle glaucoma patients, TADA was lower. In both wide and narrow-angle glaucoma cases, MDA and vitamin E levels were found to be higher. In the lower visual acuity group, MDA and vitamin E increased and SOD decreased, while in the higher IOP group, TADA was found to be significantly lower. Higher Laplace and c/d values significantly influenced TADA and vitamin E. Vitamin E demonstrated extremely higher levels in both genders, all ages, IOPs, and age groups, as well as in patients manifesting bilateral c/d abnormalities. In addition to having extremely higher decreases in TADA levels in 40- to 60-year-old patients and normal IOPs, and wide-angle glaucomas, and extremely higher increases in MDA concentrations in age-matched cases, normal visual acuities and higher IOPs were observed (Table 4 and Table 5). Furthermore, vitamin E showed extremely higher levels in all MD and PSD groups, and thicker cornea groups as well. In addition, vitamins E and A were found to be extremely higher in groups of patients with increased Laplace values. Significant differences were observed in MDA, vitamin E, SOD, and TADA levels in groups with higher MD and PSD values (Table 6 and Table 7).

**Table 2:** Variability of serum oxidative stress markers based on biochemical parameters.

**Parameters**	**Groups**	**TADA**	**AOPP**	**SOD**	**GPX**	**MDA**	**SER**	**TF**	**VİT A**	**VİT E**
Cholesterol	H (n=13)	1.63±0.09	23±7.38	3.03±0.67	1.97±0.67	1.34±0.27	28.9±12.2	276.92±72.15	0.67±0.35	17.4±4.87
	I (n=44)	1.62±0.12	34.87±30.15	3.06±0.83	1.91±0.63	1.46±0.97	37.7±13.25	259.93±59.68	0.9±0.58	20.58±8.65
	N (n=103)	1.63±0.11	31.24±16.31	3.03±0.83	2.04±0.75	1.33±0.3	37.12±13.82	280.62±84.05	0.88±0.62	20.89±15.16
Glucose	H (n=8)	1.58±0.19	28.98±13.58	3.25±1.13	2.23±0.34	1.31±0.21	42.34±6.55	264.8±55.04	0.66±0.23	20.83±8.13
	I (n=32)	1.64±0.1	36.15±25.82	3.19±0.89	1.97±0.64	1.26±0.23	34.72±10.11	270.52±59.16	0.87±0.54	20.77±12.88
	N (n=120)	1.62±0.11	31.17±25.38	2.98±0.76	1.91±0.7	2.37±9.8	37.44±14.75	267.72±75.83	0.87±0.61	20.2±10.55
Trygliceride	H (n=17)	1.62±0.17	53.12±32.17	3.24±1.11	0.7±0.58	1.25±0.3	39.29±10.4	277.94±52.53	0.86±0.44	21.85±7.75
	I (n=36)	1.63±0.08	29.1±14.79	2.95±0.75	1.88±0.7	1.42±0.27	32.13±12.53	250.58±61.66	0.83±0.47	17.99±5.43
	N (n=107)	1.63±0.1	30.61±25.56	3.12±0.78	1.97±0.67	1.31±0.26	36.67±14.5	275.11±78.05	0.79±0.5	19.49±11.05
Protein	I (n=19)	1.61±0.12	37.56±27.37	3.18±0.97	2.23±0.63	1.37±0.22	36.02±9.01	315.78±103.32	0.69±0.34	17.24±4.55
	N (n=139)	1.63±0.11	31.18±24.65	3.07±0.79	1.9±0.67	1.4±0.86	36.17±14.23	262.48±65.6	0.82±0.51	19.46±10.48
	D (n=2)	1.68±0.1	51.16±1.02	2.63±0.78	1.35±0.03	1.48±0.22	20.7±6.79	268±98.99	0.62±0.11	16.8±2.54
Albumin	I (n=3)	1.65±0.05	40.14±21.55	2.89±1.04	2.43±0.22	1.49±0.1	37.57±13.08	448±225.8	0.55±0.22	16.37±6.3
	N (n=141)	1.63±0.11	32.16±25.17	3.08±0.83	1.16±1.92	2.18±9.11	35.2±13.66	264.35±63.67	0.77±0.45	18.89±9.77
	D (n=16)	1.62±0.12	38.07±26.81	3.26±0.6	2.42±0.8	1.23±0.18	42.12±13.43	279.44±65.67	1.18±0.72	22.99±10.98

TADA: total antioxidant capacity, AOPP: advanced oxidation protein products, SOD: superoxide dismutase, Gpx: glutathione peroxidase, MDA: malonyl dialdehyde, Ser: Serine, TF: transferrine, Vit A: vitamin A, Vit: E: vitamin E, H: Very high I: Increased, D: Decreased, N: Normal, n: number.

**Table 3:** Statistical significance of serum oxidative stress markers based on biochemical parameters.

**Parameters**	**Groups**	**TADA**	**AOPP**	**SOD**	**Gpx**	**MDA**	**Ser**	**TF**	**Vit A**	**Vit E**
Cholesterol	H					*				**
	I	***		*	*	***	*		*	***
	N	**		*		**		*		**
Glucose										
	H						**			*
	I	*				*		*		***
	N	***		*	*	***	*			***
Triglyceride	H					*	*	*		*
	I					***				***
	N	*				*		*		***
Protein	I	*				***	***	*		***
	N	***			*	***				***
	D					*				
Albumin	I								*	***
	N					*				
	D	***				***				***

TADA: total antioxidant capacity, AOPP: advanced oxidation protein products, SOD: superoxide dismutase, Gpx: glutathione peroxidase, MDA: malonyl dialdehyde, Ser: Serine, TF: transferrine, Vit A: vitamin A, Vit: E: vitamin E, H: Very high I: Increased, D: Decreased, N: Normal, n: number., *:p<0.01 moderately significant,**: p<0.005 very significant, ***: p<0.001 extremely significant.

**Table 4:** Variability of serum oxidative stress markers based on clinical parameters.

**Parameters**	**Groups**	**TADA**	**AOPP**	**SOD**	**GPX**	**MDA**	**SER**	**TF**	**VIT A**	**VIT E**
Gender	F (n=106)	1.63±0.11	29.93±25.03	3.08±0.76	1.98±0.72	1.43±0.96	36.26±14.14	269.55±62.68	0.78±0.45	19.04±10.79
	M (n=54)	1.63±0.11	37.73±24.51	3.12±0.91	1.87±0.56	1.31±0.26	35.44±12.95	269.11±89.68	0.87±0.57	19.69±7.85
Age (years)	<40 (n=24)	1.66±0.11	30.44±14.72	2.75±0.67	2.04±0.74	1.34±0.35	39.69±14.61	303.21±104.34	0.74±0.38	18.16±8.02
	40–60 (n=92)	1.63±0.11	31.9±28.26	3.18±0.82	1.96±0.7	1.34±0.26	36.31±14.26	267.54±62.11	0.84±0.56	20.22±11.25
	>60 (n=34)	1.63±0.09	34.03±26.02	3.08±0.86	1.8±0.59	1.29±0.26	33.38±11.63	259.33±69.8	0.78±0.35	18.24±6.87
Visual acuity	N (n=138)	1.63±0.11	31.99±26.76	3.12±0.84	1.9±0.69	1.33±0.28	37.21±13.44	275.69±74.65	0.81±0.5	20.01±10.5
	D (n=22)	1.63±0.09	33.39±20.45	2.95±0.67	2.08±0.63	1.32±0.25	30.15±14.91	244.95±54.64	0.78±0.41	16.16±4.61
IOP	N (n=105)	1.64±0.1	31.17±21.2	3.06±0.82	1.96±0.73	1.29±0.29	35.82±11.17	270.69±77.97	0.82±0.48	19.65±11.02
	U (n=25)	1.64±0.11	28.31±13.95	3.25±0.89	1.8±0.66	1.37±0.22	35.78±15.12	273.18±74.05	0.8±0.56	20.38±9
	B (n=30)	1.61±0.1	38.7±42.96	3.02±.69	1.96±.54	1.4±0.24	37.76±12.98	272.1±60.96	0.78±0.47	17.97±6.88
C/d	N (n=89)	1.63±0.1	37.34±32.53	3.07±0.73	1.74±0.54	1.35±0.29	37.43±14.4	272.46±70.2	0.83±0.47	19.83±8.5
	U (n=26)	1.65±0.1	30.02±14.12	3.01±0.95	1.87±0.71	1.32±0.22	32.73±13.92	295.67±105.5	0.92±0.63	21.84±17.45
	B (n=45)	1.63±0.12	25.93±13.99	3.22±0.89	2.25±0.77	1.29±0.27	35.65±12.98	255.07±51.4	0.72±0.45	17.68±6.47
Angle	Wide (n=140)	1.67±0.1	31.67±0.1	3.01±0.88	1.93±0.68	1.31±0.19	35.23±12.89	285.5±81.96	1.25±0.84	29.08±25.01
	Narrow (n=20)	1.63±0.11	31.64±25.36	3.12±0.81	2.17±0.82	1.33±0.28	52.31±17.72	271.54±72.54	0.78±0.44	18.88±7.71
Drugs	(-) (n=49)	1.63±0.1	30.97±25.85	3.06±0.8	1.9±0.61	1.34±0.28	37.01±14.14	286.22±83.19	0.74±0.47	18.94±8.14
	PG (n=43)	1.67±0.09	41.23±36.53	3.15±0.71	1.83±0.74	1.29±0.27	38.13±14.58	256.36±53.32	0.97±0.56	21.99±14.85
	b-b (n=13)	1.62±0.13	24.88±12.79	3.01±0.94	1.82±0.74	1.37±0.25	33.86±16.76	248.65±68.93	0.91±0.6	19.99±9.97
	2 drugs (n=44)	1.61±0.07	32.99±19.38	3.1±0.77	2.26±0.77	1.25±0.3	31.59±9.18	245.67±37.29	0.71±0.35	17.58±7.6
	3 drugs (n=11)	1.57±0.18	35.32±21.43	3.73±1.08	2.35±0.87	1.32±0.21	36.58±7.93	295.17±85.6	0.87±0.13	17.63±3.1
Px	(+) (n=21)	1.62±0.11	31.23±8.93	3.06±0.7	1.94±0.89	1.4±0.3	29.82±12.2	249±52.52	0.688±0.19	15.54±3.29
	(-) (n=139)	1.67±0.08	32.98±18.38	2.56±0.81	2.23±0.13	1.35±0.23	28.63±5.71	217.33±44.66	0.61±0.34	16.9±6.41

TADA: total antioxidant capacity, AOPP: advanced oxidation protein products, SOD: superoxide dismutase, Gpx: glutathione peroxidase, MDA: malonyl dialdehyde, Ser: Serine, TF: transferrine, Vit A: vitamin A, Vit: E: vitamin E, U: Unilateral, B: Bilateral, D: Decreased, N: Normal, PG: Prostaglandin analogous, β-b: β-blocker, n: number.

**Table 5:** Statistical significance of serum oxidative stress markers based on clinical parameters.

**Parameters**	**Groups**	**TADA**	**AOPP**	**SOD**	**Gpx**	**MDA**	**Ser**	**TF**	**Vit A**	**Vit E**
Gender	Female	**				**				***
	Male	**				**			*	***
Age (years)	<40	*		***		*		*		***
	40–60	***				***		*		***
	>60				*	*				***
Visual acuity	N	*				***	*	*		***
	D			*		*				*
IOP	N	***		*		*	*		*	***
	U	*				***				***
	B			*		***	*			***
c/d	N	***		*	***	***	*	*		
	U					**		*		**
	B	*			***	*				***
Angle	Wide	***			*	***		*		***
	Narrow					*	***		*	***
Drugs	(-)			*	*	***		*		***
	PG	*				*			*	**
	β-b			*		**				**
	2 drugs									
	3 drugs									
Px	(+)		*			***				**
	(-)				*	**				*

TADA: total antioxidant capacity, AOPP: advanced oxidation protein products, SOD: superoxide dismutase, Gpx: glutathione peroxidase, MDA: malonyl dialdehyde, Ser: Serine, TF: transferrine, Vit A: vitamin A, Vit: E: vitamin E, U: Unilateral, B: Bilateral, D: Decreased, N: Normal, PG: Prostaglandin analogous, β-b: β-blocker, n: number, *:p<0.01 moderately significant,**: p<0.005 very significant, ***: p<0.001 extremely significant.

**Table 6:** Variability of serum oxidative stress markers based on visual field, pachymetric parameters and laplace scores.

**Parameters**	**Groups**	**TADA**	**AOPP**	**SOD**	**GPX**	**MDA**	**SER**	**TF**	**VIT A**	**VIT E**
MD	N (n=107)	1.64±0.1	31±25.18	3.03±0.85	1.9±0.73	1.31±0.28	36.8±15.02	266.7±63.2	0.88±0.54	20±8i77
	U (n=27)	1.59±0.16	48.05±24.16	3.22±0.9	2.6±0.68	1.35±0.21	40±13.68	278.08±64.49	1.61±0.76	26.7±22.97
	B (n=26)	1.62±0.12	26.25±9.26	3.22±0.79	1.88±0.48	1.22±0.3	42.4±19.62	283.73±58.29	0.84±0.41	20.51±8.58
PSD	>10 (n=112)	1.61±0.12	24.77±10.36	3.29±0.91	2.07±0.49	1.37±0.24	39.39±13.5	289.71±70.67	0.95±0.68	22.64±17.92
	<10 (n=48)	1.64±0.1	34.6±27.02	3.03±0.82	1.97±0.78	1.28±0.29	38.07±15.48	263.43±58.28	0.9±0.54	20.49±9.04
Corneal thickness	H (n=32)	1.63±0.06	32.2±12.69	2.92±0.66	1.72±0.75	1.36±0.3	26.72±3.14	238.2±69.67	0.64±0.21	15.48±1.26
	N (n=68)	1.64±0.1	29.65±17.47	3.3±0.72	2.07±0.8	1.24±0.28	39.8±14.91	262.04±52.67	0.86±0.58	21.61±18.8
	L (n=60)	1.61±0.12	33.35±31.53	3.02±0.81	1.83±0.76	1.22±0.29	31.13±14.23	262.71±52.24	0.98±0.59	20.44±9.3
Laplace value	I (n=76)	1.64±0.07	31.01±19.18	3.03±0.55	1.82±0.43	1.32±0.31	36.54±21.05	266.22±77.98	0.79±0.32	18.18±7.79
	D (n=84)	1.68±0.08	36.25±24.13	3.65±0.86	2.66±0.58	1.19±0.1	40.47±11.42	289.11±44.84	1.55±0.74	31.05±10.95

TADA: total antioxidant capacity, AOPP: advanced oxidation protein products, SOD: superoxide dismutase, Gpx: glutathione peroxidase, MDA: malonyl dialdehyde, Ser: Serine, TF: transferrine, Vit A: vitamin A, Vit: E: vitamin E, H: Very high I: Increased, D: Decreased, N: Normal, n: number.

**Table 7:** Statistical significance of serum oxidative stress markers based on visual field, pachymetric parameters and laplace scores.

**Parameters**	**Groups**	**TADA**	**AOPP**	**SOD**	**Gpx**	**MDA**	**Ser**	**TF**	**Vit A**	**Vit E**
MD	N	*		*		***	*			***
	U					**	*		*	***
	B									***
PSD	>10	*		**		***		*	*	***
	<10	**				*	*		*	***
Corneal thickness (μm)	H					*				*
	N									
	L	**							*	***
Laplace value	I	*						*	***	***

TADA: total antioxidant capacity, AOPP: advanced oxidation protein products, SOD: superoxide dismutase, Gpx: glutathione peroxidase, MDA: malonyl dialdehyde, Ser: Serine, TF: transferrine, Vit A: vitamin A, Vit: E: vitamin E, H: Very high I: Increased, D: Decreased, N: Normal, n: number, *:p<0.01 moderately significant,**: p<0.005 very significant, ***: p<0.001 extremely significant.

When compared with the normal control groups, MDA levels in the group of patients with hyperglycemia, px, and Gpx levels, in cases with bilaterally higher c/d, ratios were found to be extremely higher and lower, respectively. In addition, in the group with lower visual acuity, MDA and vitamin E levels were moderately higher and SOD levels were moderately lower when compared to the patients with normal visual acuities. Vitamins E and A were found to be significantly increased in the group with higher IOP Laplace scores as well.

## Discussion

Glaucoma, being a nonsystemic disease, is the most frequent etiology of irreversible blindness worldwide is not only an ocular pathology [13]. However, studies related to its effects on serum oxidative stress markers are quite limited in the existing literature. In ocular pathologies, such as Behçet’s disease [18] and cataract [19], the serum oxidant/antioxidant profile was reportedly altered. However, in glaucoma, it was determined that the serum glutathion concentration decreased [20], and composition of blood fatty acids were altered [21]. In this study, the effects of 5 biochemical and 12 clinical parameters on serum levels of oxidation degradation products and 8 antioxidants were examined.

In the main comparison between the patient and control groups, the observation of significant differences in all data (excluding AOPP) should be emphasized. In this study, TADA, protein oxidation end product AOPP, and the enzymes SOD and Gpx, were found to be decreased. On the other hand, lipid oxidation end product MDA, Ser and TF, and liposoluble vitamins A and E increased in the patient group. Glaucoma is an optic neuropathy, and oxidative stress plays an important role in its etiopathogenesis. Thus, adipose tissue damage is its predominant characteristic [5]. Oxidative reactions occurring within tissues lead to the formation of different end-products, according to the molecules they affect. Among these end-products that can be traced in serum, AAOP is formed as a result of protein degradation [22]. Further, the increments in levels of vitamin E and MDA in the patient group were found to be extremely significant. Vitamins A and E are lipid soluble [23], and MDA is a lipid oxidation degradation product [24]. In glaucoma patients, MDA increases twofold in the anterior chamber [25], and in cases with cataract, its serum levels are also enhanced [19]. Since retina and nervous tissue are rich in lipid, these results are significant. In addition, in the hypertriglyceridemia group, TADA, Gpx, and SOD were statistically significantly lower, and TF, MDA, and vitamin E were statistically significantly higher. On the other hand, in the hypercholesterolemia group, significant differences in all parameters, except AOPP, were observed, which demonstrates the importance of lipid metabolism in the pathogenesis of glaucoma. This is also consistent with previous findings [21]. Addtitionally, the most significant and consistent results were observed for all parameters in the levels of vitamin E. However, in intergroup comparisons between MDA and TADA, as well as with the control group, the most frequently encountered significant elevations were noted for oxidative stress indicators.

When compared with the control group, vitamin E was significantly elevated in all patients except for those with normal corneal thickness, lower Laplace scores, normal c/d, px(-), and hypoproteinemia. However, lipid-soluble antioxidant vitamin A only exhibited extremely higher levels in patients with higher Laplace scores. These results emphasize vitamin E as an antioxidant and a neurohormone.

It is recognized that oral or parenteral vitamin E accumulates more frequently inside the retina [26]. A study conducted on human eyes reported that retinal vitamin E levels were higher than those found in choroidal and vitreal tissues, and were directly proportional to serum vitamin E levels [27]. In animal trials, oral or parenteral administration of 100 mg/kg dl-α-tocopheril acetate were reported to similarly induce 3–6 fold increases in plasma levels, while after oral intake, retinal and vitreal levels were achieved later [28]. Apart from other antioxidants, vitamin E contains sensitive mechanisms for the regulation of tissue surface. Discovery of the tocopherol transfer protein, specific membrane receptors, and cytosolic transfer proteins, has suggested that this molecule might have other functions apart from its antioxidant effects. Indeed, many in vivo and in vitro studies performed in normal and neoplastic cells have demonstrated that α-tocopherol had specific effects, including gene regulation [29]. We have specific evidence that this phenomenon also applies to ocular tissues [30]. Again, it has been established that some vitamin E derivatives act as neurohormones, and initiate various intracellular conduction pathways with a lock –and-key model. Cell culture and animal studies have confirmed that, among these specific effects, those on PKC were unique for PKC, and closer isomers (e.g., β-tocopherol), and distinct antioxidants (e.g., probucol), lacked these effects [31]. Various trials have reported that retinal vascular dysfunction secondary to hyperglycemia is corrected by α-tocopherol through the DAG-PKC pathway [32].

Regarding glaucoma, the PKC pathway also has significant effects on non-vascular ocular smooth muscles, including trabecular meshork of the eye [33], PGF_2α_, and matrix metalloproteinases [34]. In addition, some publications have stated that glutamine transporter activity, which plays a key role in neurodegeneration, is regulated by PKC [35]. Retinal blood flow regulating and neuroprotective effects of α-tocopherol in glaucoma patients have been clinically demonstrated [10]. Moreover, α-tocopherol is recognized for prolonging life span in retinal cell cultures [36]. Taking all of these issues into consideration, significant and consistent elevation manifested by vitamin E, which has a special role among antioxidants [8] in glaucoma patients, is very important.

Elevations in Ser in the hyperproteinemia group were found to be extremely significant. Ser is an aminoacid that plays an important role in the antioxidant and neuromodulator effects of vitamin E [37]. Another important point is that PKC, which interacts with α-tocopherol, is a type of Ser/Treonin kinase [38].

Another elevated liposoluble vitamin was vitamin A. Similar to tocopherols, vitamin A derivatives are also known to be neuromodulators in the retina [39], to improve endothelial function by reducing the concentration of reactive oxygen species in the vessel wall [40], and possess specific receptors in the nervous system [41].

In addition, Vitamin E and MDA elevations observed in the px(+) group (which was more significant than that of px(-) group, but meaningful relative to the control group) are consistent with the literature. The study performed by Yılmaz et al., which demonstrated that MDA levels in cataract patients with px were higher when compared with other forms of cataract, has been among trials indicating that an ocular pathology could affect serum oxidative stress markers [42].

In this study, clinical findings on the effects of glaucoma on oxidative stress indicators have been demonstrated to shed light on the pathogenesis of glaucoma. The association between glaucoma and lipid oxidation has been revealed as well. Another point to be emphasized is that, in addition to being an antioxidant, vitamin E has unique neurohormone- like activities and regulatory mechanisms, and its serum levels increase in conjunction with the severity of clinical findings of glaucoma. Further studies are needed to conclude that this molecule is the sole predictor of glaucoma. However, based on the results of this study, the significance of vitamin E as a neuroprotective agent with neurohormone-like activities, independent to being an antioxidant, has been revealed once more.
